# Can low-dose abdominal CT replace abdominal plain film in evaluation of acute abdominal pain?

**DOI:** 10.3109/03009730903294871

**Published:** 2010-04-07

**Authors:** Olle Haller, Lars Karlsson, Rickard Nyman

**Affiliations:** ^1^Department of Diagnostic Radiology, Gävle Hospital, GävleSweden; ^2^Department of Surgery, Gävle Hospital, Gävle; ^3^Department of Diagnostic Radiology, Uppsala University Hospital, UppsalaSweden

**Keywords:** Abdominal pain, computed tomography, radiation dosage

## Abstract

**Background:**

Non-contrast computed tomography (NCT) has become an important diagnostic tool in acute abdominal pain, but the drawback is the increased radiation dose compared to abdominal plain film (APF).

**Purpose:**

To evaluate whether NCT, including low-dose computed tomography (LDCT, using 50 mAs), provides more diagnostic information than APF in patients presenting with acute non-traumatic abdominal pain and if the use of CT can reduce the total number of additional radiograms. A second aim was to compare the diagnostic outcome between standard-dose computed tomography (SDCT) and LDCT.

**Material and methods:**

During 2000, 2002, and 2004 a total of 222 patients were retrospectively reviewed, and 86 patients had APF, 60 had SDCT, and 76 had LDCT. The radiological report of each patient was compared with the final diagnosis obtained from the medical record within 30 days. Additional radiograms were registered, and a total radiation dose excluding or including APF or NCT was calculated.

**Results:**

NCT gave a correct diagnosis in 50%, compared to 20% with APF (*P* < 0.001). The total number of additional radiograms was substantially lower in the computed tomography (CT) group compared to the APF group (*P* < 0.001), and the average sum of radiation dose was similar for APF and LDCT.

**Conclusion:**

NCT was found to be significantly better at providing diagnostic information than APF in patients presenting with acute abdominal pain. It reduced the number of additional radiograms, but the total patient dose remained somewhat higher in the CT group even when using LDCT with 50 mAs.

## Introduction

Computed tomography (CT) has, particularly since the introduction of spiral CT, become more and more useful in the evaluation of acute abdominal pain. CT is known to have a better diagnostic accuracy than abdominal plain film (APF) (1–3), and when a patient is presenting with acute abdominal pain, non-contrast CT (NCT), without intravenous or peroral contrast, has been found to give reliable information (4).

Several studies have shown the value of NCT in the evaluation of acute appendicitis, diverticulitis, and cholecystitis with a high sensitivity and specificity (5–7). Abdominal NCT is also as sensitive as APF in diagnosing ileus (8,9) and more sensitive than APF in detecting intraperitoneal free air (10). NCT has become an alternative to intravenous urography in the evaluation of renal colic with a very high sensitivity and specificity for the detection of ureteral stones (11). CT is also as effective as urography in recognizing signs of ureteral obstruction (12) and is able to detect additional urinary tract findings (13) as well as alternative non-genitourinary diagnoses (14).

NCT have been suggested to replace APF in patients with acute abdominal pain (15,16) as CT has been found to give a superior diagnostic sensitivity of 96% compared to 30% for APF (17). Nevertheless this has been (18) and still is a controversial issue (19), and the increased radiation dose caused by the CT examination has been of major concern (20). However, it is possible to reduce the radiation dose with maintained acceptable image quality (21). Attempts have been made to decrease the radiation dose of the CT examination, mainly in the evaluation of renal colic, by reducing the tube current time product from 120–260 mAs (milliampere seconds) to 30–76 mAs. This so-called low-dose CT (LDCT) has been shown in several studies to have a sensitivity and specificity above 90% in the detection of ureteral stones (22). Even an extreme LDCT with 6.9 mAs, giving a radiation dose equal to an APF, has been found to be adequate for diagnosing ureteral stones (23). If the CT examination provides more information than the APF or a urography the total number of examinations performed might also be reduced, which can give a further total dose reduction (24).

In our department NCT was introduced in 2001 instead of APF in patients with acute abdominal pain. Initially a tube current time product of 120 mAs was used, and in 2003 a reduction to 50 mAs was made.

The aim of the study was to evaluate whether NCT including LDCT can provide more diagnostic information and reduce the need of additional radiograms compared with APF in patients with acute abdominal pain. A second aim was to compare the diagnostic outcome between standard-dose CT (SDCT) and LDCT.

## Material and methods

In advance of the study, an application to the local ethics committee was made, and the committee's response was that this kind of retrospective study did not need approval of an ethics committee. However, the local ethics committee had recommendations regarding patient integrity, which were followed. Written informed consent was obtained from the patients to get access to their medical records.

Patient selection was made using the journal system of the department of radiology where patients were referred from the department of surgery and examined with APF or NCT. Since our aim was to compare the methods APF, SDCT, and LDCT we chose to include patients from three time periods when each one of the modalities was used as the routine method. For this reason patients from the months January, February, and March in the years 2000, 2002, and 2004 were included in the study.

The indication for radiologic examination was acute abdominal pain of unknown cause. According to written guide-lines from the department of radiology, NCT was supposed to be performed using the same indications as earlier APF. The intention was that patients in whom a specific diagnosis like abscess, aortic aneurysm, gall-stone, or appendicitis was strongly suspected would be examined with another method like ultrasound or contrast-enhanced CT. According to the patients' journals there was a clinical suspicion of free abdominal gas, ileus, or both in 91% of the APF patients and in 80% of the NCT patients. In 3.5% of the APF patients and 6% of the NCT patients ureteral stones was the most suspected condition. Of the remaining patients a majority were just described as ‘abdominal pain’.

A total of 225 patients fulfilled the inclusion criteria with symptoms of acute non-traumatic abdominal pain, examination within 72 hours from admission, age of 18 years or older, and no operations or imaging studies performed the last 2 weeks before the radiological examination. Three patients were found to have been examined with intravenous or peroral contrast media and were, therefore, excluded. Thus a total of 222 patients (109 males and 113 females with mean age of 66 years, range 18–92 years), were included in the study (Table I).

**Table I.** The number of included patients examined with the different modalities during January, February, and March in the years 2000, 2002, and 2004.

Method	2000	2002	2004	Total
Abdominal plain film	82	2	2	86
Standard-dose computed tomography	0	45	15	60
Low-dose computed tomography	0	0	76	76

A total of 86 patients had APF (39 males and 47 females with mean age of 69 years, range 28–92 years).

In 2004 the intention was that all patients examined with CT were going to have LDCT. However, the radiographer had to reduce the dose manually in these patients, and this was accidentally forgotten in 15 out of 91 patients. Since these patients now had gone through a SDCT they were instead included in the SDCT group.

Thus a total of 60 patients had SDCT (31 males and 29 females with mean age of 67 years, range 28–87 years), while 76 had LDCT (39 males and 37 females with mean age of 61 years, range 18–89 years). The mean age of the total CT group was 64 years.

CT scans were obtained with a four-detector row Siemens Volume Zoom (Siemens Medical Solutions, Erlangen, Germany). The SDCT examinations were performed with 80–165 mAs, 120 kV (kilovolts), collimation 4 × 2.5 mm, feed 12.5 mm, and pitch 1.3. In LDCT 30–50 mAs, 140 kV, collimation 4 × 5 mm, feed 25 mm, and pitch 1.3 were used. All CT examinations were reconstructed with axial images with a 5-mm interval.

APF images were obtained with a Philips Bucky Diagnost TH (Philips Medical Systems, Best, the Netherlands). All APF examinations included four radiograms, two anterior-posterior projections (35 × 43 cm cassette, 16 mAs, 77 kV) of the upper and lower abdomen, one horizontal, anterior-posterior projection (35 × 43 cm cassette, 16 mAs, 81 kV) with the patient in a left lateral decubitus position, and finally a free gas projection (24 × 30 cm cassette, 20 mAs, 60 kV) of the right hemidiaphragm, also with the patient in a left lateral decubitus position. All reports were primarily either written or double-checked by a specialist in radiology.

The diagnoses in the X-ray reports were retrospectively reviewed and compared with the diagnoses in the medical records at the department of surgery. The final diagnosis written in the medical record within 30 days of the radiological examination was regarded as gold standard. The final diagnosis was confirmed operatively in 58 patients, by other radiological methods in 49 patients, and by endoscopy in 10 patients. In 11 patients the final diagnosis was an already known disease, mostly a known malignancy. In 44 patients the diagnosis remained unclear even after 30 days. In the remaining 50 patients (21% of the APF patients and 24% of the NCT patients) the diagnosis was received by using the information of the original APF or CT examination together with chemical analyses and clinical outcome. The most common diagnosis in this group was constipation (19 patients). All patients were treated at the same department of surgery.

All X-ray reports and matching final diagnoses were reviewed together with a specialist in surgery, and the impact of each X-ray report was graded into one of four groups named A, B, C, and D. Group A included patients were the diagnosis in the X-ray report was in complete agreement with that in the medical record. Reports that led to an immediate therapeutic step (free abdominal gas) were also included in this group. Group B included patients where the findings in the X-ray report were valuable but did not lead to a direct diagnosis. The report provided useful information but further radiological, clinical, or laboratory examinations had to be made to confirm the diagnosis. Patients with a known disease, most often a malignancy, where the report showed status quo, were also included in this group. The radiological report did not provide any new diagnostic information in these cases, but the information about the state of the known disease was still regarded as valuable. Group C included patients where the radiological report did not lead to a correct diagnosis. Finally, Group D included patients where the final diagnosis remained unclear even when the patient left the hospital, and most of these were diagnosed as ‘unspecific abdominal pain’ in the medical report.

All additional radiograms, divided into abdominal and non-abdominal radiograms, within 30 days from the original examination were registered for each patient. A total patient dose was calculated by using the average dose of each radiogram that is reported to the Swedish Radiation Protection Authority (Table II).

**Table II.** Effective dose in millisievert (mSv) for different radiograms reported to Swedish Radiation Protection Authority.

Radiograms	Effective dose (mSv)
Abdominal plain film	1.3
Standard dose CT abdomen	7.3
Low dose CT abdomen	4.2
Chest X-ray	0.1
Urography	1.3
Lumbar spine X-ray	1.6
Neck X-ray	0.4
Shoulder X-ray	0.01
CT abdomen unenhanced and enhanced	10.5
CT thorax	2.8
CT skull	2.3
CT urography	7.7
Colon enema, double-contrast	8.2

Pearson's chi-square test was used to evaluate the diagnostic results, expressed in percentage, of the different radiological methods in the different diagnostic groups A, B, C, and D. Non-parametric variables like the number of additional radiograms and the average patient doses were analysed with the non-parametric test of Mann-Whitney.

## Results

The diagnoses made with APF as well as the final diagnoses of the patients in this group are presented in Table III, and the diagnoses of the SDCT and LDCT groups are presented in Table IV and Table V.

**Table III.** Radiologic and final diagnoses of the patients examined with abdominal plain film (APF).

Diagnosis group	Radiologic diagnosis	Final diagnosis	Patients, *n*
A: Diagnostic report	Ileus, volvulus	Ileus, volvulus	8
	Free gas	Perforation	3
	Obstipation	Obstipation	5
	Ascites	Ascites	1
B: Report valuable but not diagnostic	Slight bowel dilation	Obstipation	1
	Gastric retention	Gastritis, gastric hernia	1
	Pancreas calcifications	Pancreatitis	1
	Kidney stone (ureteral stone not described)	Ureteral stone	1
	Ascites	Ascites and malignancy	1
	Dilated bowel, with or without metastases	Colon or rectal cancer	3
	Known pathology, status quo	Known pathology, status quo	1
C: Report not diagnostic	No conclusive finding	Perforation	1
	No conclusive finding	Appendicitis	3
	No conclusive finding	Diverticulitis, colitis	9
	No conclusive finding	Obstipation	4
	No conclusive finding	Gastric ulcer	1
	No conclusive finding	Gastroenteritis	2
	No conclusive finding	Bowel gangrene	2
	No conclusive finding	Cholecystitis	6
	No conclusive finding	Pancreatitis	4
	No conclusive finding	Liver cirrhosis	2
	No conclusive finding	Urinary infection	1
	No conclusive finding	Urine retention	1
	No conclusive finding	Malignancy	5
	No conclusive finding	Cardiac disease	1
	Obstipation	Gall-stone	1
	Obstipation	Pneumonia	1
D: Clinically unclear diagnosis	No findings	Unspecific abdominal pain	12
	Obstipation	Unspecific abdominal pain	4
Total			86

**Table IV.** Radiologic and final diagnoses of the patients examined with standard-dose computed tomography (SDCT).

Diagnosis group	Radiologic diagnosis	Final diagnosis	Patients, *n*
A: Diagnostic report	Ileus, volvulus	Ileus, volvulus	4
	Free gas	Perforation	4
	Obstipation	Obstipation	2
	Ascites	Ascites	2
	Diverticulitis, colitis	Diverticulitis, colitis	2
	Hernia	Hernia	1
	Invagination	Invagination	1
	Gall-stone, cholecystitis	Gall-stone, cholecystitis	2
	Ureteral stone, hydronephrosis	Ureteral stone, hydronephrosis	3
	Previously unknown malignancy	Previously unknown malignancy	1
	AAA (abdominal aortic aneurysm)	AAA, acute operation	1
B: Report valuable but not diagnostic	Slight bowel dilation	Ileus	1
	Gastric retention	Gastritis, gastric hernia	2
	Gastroenteritis?	Gastroenteritis	1
	Ascites	Bowel gangrene	1
	Gall-stone	Cholecystitis	1
	Ascites	Ascites and malignancy	2
	Dilated bowel, with or without metastases	Colon or rectal cancer	1
	Known pathology, status quo	Known pathology, status quo	2
C: Report not diagnostic	No conclusive finding	Ileus	1
	No conclusive finding	Diverticulitis, colitis	1
	No conclusive finding	Obstipation	1
	No conclusive finding	Gastric ulcer	1
	No conclusive finding	Gastroenteritis	1
	No conclusive finding	Cholecystitis	1
	No conclusive finding	Urinary infection	2
	Obstipation	Gall-stone	1
	Ascites	Obstipation	1
D: Clinically unclear diagnosis	No findings	Unspecific abdominal pain	12
	Obstipation	Unspecific abdominal pain	4
Total			60

**Table V.** Radiologic and final diagnoses of the patients examined with low-dose computed tomography (LDCT).

Diagnosis group	Radiologic diagnosis	Final diagnosis	Patients, *n*
A: Diagnostic report	Ileus, volvulus	Ileus, volvulus	7
	Free gas	Perforation	3
	Obstipation	Obstipation	7
	Ascites	Ascites	1
	Diverticulitis, colitis	Diverticulitis, colitis	7
	Appendicitis	Appendicitis	2
	Pancreatitis	Pancreatitis	3
	Gall-stone, cholecystitis	Gall-stone, cholecystitis	3
	Ureteral stone, hydronephrosis	Ureteral stone, hydronephrosis	5
	Previously unknown malignancy	Previously unknown malignancy	2
	AAA (abdominal aortic aneurysm)	AAA, acute operation	1
	Abscess	Abscess	1
	Haematoma	Haematoma	2
	Hernia	Hernia	1
B: Report valuable but not diagnostic	Slight bowel dilation	Ileus	2
	Slight bowel dilation	Obstipation	1
	Pancreatitis	Cholecystitis	1
	Ascites	Ascites and malignancy	2
	Dilated bowel, with or without metastases	Colon or rectal cancer	1
	Mesenteric artery calcification	Ischaemic bowel disease	1
	Known pathology, status quo	Known pathology, status quo	5
C: Report not diagnostic	No conclusive finding	Ileus	1
	No conclusive finding	Obstipation	2
	No conclusive finding	Cholecystitis	1
	No conclusive finding	Cardiac disease	1
	Gastric retention	Multiorganic failure	1
D: Clinically unclear diagnosis	No findings	Unspecific abdominal pain	10
	Obstipation	Unspecific abdominal pain	2
Total			76

The percentage of patients distributed between the different diagnostic groups A, B, C, and D for the radiological methods APF, SDCT, and LDCT are presented in Table VI. The percentage of CT with correct diagnosis was significantly higher than APF and independently of whether group D was included or not (*P* < 0.001). In group A, CT (SDCT and LDCT) was found to give the correct diagnosis in 50% (95% confidence interval (CI) 41.6%–58.4%) compared with 20% (95% CI 11.4%–28.2%) of the patients with APF, a difference found to be significant (*P* < 0.001). The sum of correct diagnoses for group A and B was 68% with CT and 30% with APF.

**Table VI.** The percentage of patients distributed between the groups A, B, C, D for the radiological methods abdominal plain film (APF), standard-dose computed tomography (SDCT), low-dose computed tomography (LDCT), and computed tomography (CT) total. Number of patients in parenthesis.

	A: Diagnostic report	B: Report valuable but not diagnostic	C: Report not diagnostic	D: Clinically unclear diagnosis	Total number of patients
APF	20% (17)	10% (9)	51% (44)	19% (16)	86
CT Total	50% (68)	18% (24)	12% (16)	21% (28)	136
SDCT	38% (23)	18% (11)	17% (10)	27% (16)	60
LDCT	59% (45)	17% (13)	8% (6)	16% (12)	76

There was no significant difference in diagnostic results when comparing SDCT and LDCT, independently of whether group D was included or not (*P* = 0.07 and *P* = 0.1, respectively). However, when comparing the diagnostic results in only group A, there was a significant difference in favour of LDCT (*P* < 0.02).

The average numbers of additional abdominal and non-abdominal radiological examinations are presented in Table VII. Analyses with a non-parametric test showed that SDCT and LDCT had a significantly lower number of additional abdominal radiograms than APF at 1 week, at admission, and at 30 days (*P* < 0.001). However, only LDCT showed significant reduction of non-abdominal radiograms for all three time periods (*P* < 0.04). The combined result of SDCT and LDCT (CT total) showed that 69% of the patients did not require any additional abdominal radiograms during the time of admission compared with 38% in the APF group (Table VIII). Only 4% in the CT group required two or more abdominal radiograms during admission, compared with 26% in the APF group.

**Table VII.** Average number of additional abdominal radiograms and non-abdominal radiograms (in parentheses) for the radiological methods APF, SDCT, LDCT, and CT total.

	Within 1 week	During admission	Within 30 days
APF	0.94 (0.42)	1.00 (0.50)	1.23 (0.82)
CT Total	0.33 (0.20)	0.38 (0.30)	0.61 (0.44)
SDCT	0.32 (0.28)	0.38 (0.47)	0.65 (0.63)
LDCT	0.34 (0.13)	0.37 (0.17)	0.58 (0.29)

**Table VIII.** The percentage of additional abdominal radiograms during admission for the radiological methods APF, SDCT, LDCT, and CT total. Number of patients in parenthesis.

Number of additional abdominal radiograms	0	1	2 or more	Total number of patients
APF	38% (33)	36% (31)	26% (22)	86
CT Total	69% (94)	26% (36)	4% (6)	136
SDCT	72% (43)	23% (14)	5% (3)	60
LDCT	67% (51)	29% (22)	4% (3)	76

The calculated average doses including both additional abdominal and non-abdominal radiograms for each radiological method (APF, SDCT, and LDCT), as well as the added doses of APF (1.3 mSv), SDCT (7.3 mSv), and LDCT (4.2 mSv), are presented in Table IX. The average doses for APF and LDCT including the additional radiograms were almost the same, 6.7 and 6.2 mSv, respectively. In each group there were a few patients who stood out with a lot of additional radiograms resulting in a much higher total dose, more frequently in the APF group. Since these few patients could have an important influence on the average dose in each group the results were analysed with a non-parametric test. With this test APF was found to have a significantly lower total dose even in comparison with the LDCT (*P* = 0.003 at 30 days). A large number of patients in each group were only examined by APF (38%) or NCT (69%) and did not have any additional radiograms. These patients received a higher dose if only examined with NCT instead of only APF, which explains the result of an overall significant lower dose for the APF method.

**Table IX.** Average calculated dose in millisievert (mSv) with original radiograms excluded and included (in parenthesis).

	Abdominal radiograms within 1 week	Abdominal radiograms during admission	Abdominal radiograms within 30 days	Total radiograms within 30 days
APF	3.6 (4.9)	4.0 (5.4)	5.0 (6.3)	5.3 (6.7)
SDCT	4.2 (8.5)	1.7 (9.0)	2.3 (9.5)	2.8 (10.1)
LDCT	1.2 (5.4)	1.3 (5.5)	1.9 (6.1)	2.0 (6.2)

An estimation of the cost for either APF or NCT was made using the purchase price of the equipment and its time of use, the salaries of radiographers and physicians, and their time involved in the examination. According to this calculation the cost of an APF and NCT were found to be US$47 and US$89, respectively. This calculation did not include other costs like administration, education, and buildings, which would affect the costs in a similar way for the two examinations. The main explanation for the differences in examination cost was found to be the differences in the purchase price of the equipment. The additional radiological examinations during 30 days generated a mean cost of US$491 in the APF group compared to US$248 in the NCT group.

## Discussion

This study demonstrates that NCT is significantly better in providing diagnostic information than APF in patients presenting with acute abdominal pain, as has been shown by others (17). It also reduces the number of additional radiograms, but the total radiation dose to the patient group remained somewhat higher in the CT group even when using LDCT, compared to APF. However, the results indicate that as a group the total radiation doses to the LDCT and APF groups were almost equal. The total average patient dose was calculated from the average doses of the examinations performed which gives a more crude measurement of how the total dose is affected by the choice of modality than if the doses had been calculated independently in each patient. Also the way the material was handled statistically can have an influence on the final differences in dose results. With newer equipment it seems possible to reduce the radiation dose even more by further decrease of the tube current time product, which will further increase the advantage of using CT.

The reduction of additional radiograms is an important finding and benefit of using CT. A quick and correct diagnosis with reduced use of other diagnostic tests will be of great benefit for the patients as well as for the health care system. However, an over-use of LDCT will result in increased radiation doses to the population, and the health advantages of a quick and correct diagnosis might be somewhat counteracted by the increased risk of X-ray-induced malignancy (25). This of course means less for elderly patients compared to younger patients, where indications for using LDCT must be kept very strict (26). Our results also indicate that APF can be used when bowel obstruction and constipation are the main indications. However, for diagnosing free air, APF cannot be recommended, and LDCT is now considered the method of choice (10). LDCT has its clear advantage in patients with severe acute abdominal pain with unclear address.

Surprisingly LDCT was found to provide better diagnostic results than SDCT, and the most likely explanation was that the radiologists gained more experience in interpreting unenhanced CT imaging in the acute abdomen during the 2 years until LDCT was started.

We admit that the result, using this retrospective approach, does not permit a reliable comparison between SDCT and LDCT, which was a second aim in this study. On the other hand this indicates that the differences between APF and CT will increase over time in favour of CT as experience with the method grows.

The development of new methods and exchange of old methods happen very fast today, and unfortunately these exchanges of methods are often done without being critically evaluated if it really has a health benefit. This is especially important when we change from methods with less radiation to more radiation as the clear trend of the increased use of CT is. It is important to be able to measure the health effect of such changes, and hopefully this paper can contribute to this.

The drawback with this study was that it was retrospective and, therefore, the groups not exactly comparable. There was a difference in mean age between the APF (69 years) and CT (64 years) groups. This can be due to the referring doctor changing his behaviour over the time when APF was exchanged to CT, becoming more aware that CT could be helpful in diagnosing inflammatory disease such as appendicitis, cholecystitis, and diverticulitis, not possible with APF. Especially, the increased request for diagnosing appendicitis could explain the somewhat lower mean age in the CT group. Our experience is, however, that when APF was the method of choice it was generally used as a first-step investigation in most patients with acute abdominal pain. This is illustrated by the fact that 47% (40 patients of 86) of the patients in the APF group received one of the final diagnoses appendicitis, diverticulitis, abscess, ureteral stone, cholecystitis, bowel gangrene, liver cirrhosis, aortic aneurysm, haematoma, ascites, or malignancy, compared to 39% (53 patients of 136) in the CT groups. Nevertheless, the change in behaviour over time for both referring doctor and radiologist will have an effect on the comparison between the groups.

The advantage with this retrospective study was that the different groups were extracted during a time when each method was the mainstream one.

The cost analyses demonstrated that the cost for a NCT (US$89) examination was somewhat higher compared to an APF (US$47) examination. This difference was mainly due to the differences in purchase price of the equipment. However, if all additional radiological examinations during 30 days were included, the mean cost was clearly in favour of the NCT group compared to the APF group.

The results of this study show that LDCT is more informative in the acute abdomen than APF, reduces the need of additional radiological methods, and that a low-dose approach does not necessarily impair the diagnostic quality. This makes LDCT a promising tool, and the key point in the future must be to make the radiation dose as low as possible.

## References

[1] Ahn SH, Mayo-Smith WW, Murphy BL, Reinert SE, Cronan JJ (2002). Acute nontraumatic abdominal pain in adult patients: abdominal radiography compared with CT evaluation. Radiology.

[2] Nagurney JT, Brown DF, Novelline RA, Kim J, Fischer RH (1999). Plain abdominal radiographs and abdominal CT scans for nontraumatic abdominal pain–-added value?. Am J Emerg Med.

[3] Rosen MP, Sands DZ, Longmaid HE, Reynolds KF, Wagner M, Raptopoulos V (2000). Impact of abdominal CT on the management of patients presenting to the emergency department with acute abdominal pain. AJR Am J Roentgenol.

[4] Basak S, Nazarian LN, Wechsler RJ, Parker L, Williams BD, Lev-Toaff AS (2002). Is unenhanced CT sufficient for evaluation of acute abdominal pain?. Clin Imaging.

[5] Malone AJ, Wolf CR, Malmed AS, Melliere BF (1993). Diagnosis of acute appendicitis: value of unenhanced CT. AJR Am J Roentgenol.

[6] Tack D, Bohy P, Perlot I, De Maertelaer V, Alkeilani O, Sourtzis S (2005). Suspected acute colon diverticulitis: imaging with low-dose unenhanced multi-detector row CT. Radiology.

[7] Fidler J, Paulson EK, Layfield L (1996). CT evaluation of acute cholecystitis: findings and usefulness in diagnosis. AJR Am J Roentgenol.

[8] Balthazar EJ (1994). George W. Holmes Lecture. CT of small-bowel obstruction. AJR Am J Roentgenol.

[9] Taourel PG, Fabre JM, Pradel JA, Seneterre EJ, Megibow AJ, Bruel JM (1995). Value of CT in the diagnosis and management of patients with suspected acute small-bowel obstruction. AJR Am J Roentgenol.

[10] Earls JP, Dachman AH, Colon E, Garrett MG, Molloy M (1993). Prevalence and duration of postoperative pneumoperitoneum: sensitivity of CT vs left lateral decubitus radiography. AJR Am J Roentgenol.

[11] Vieweg J, Teh C, Freed K, Leder RA, Smith RH, Nelson RH (1998). Unenhanced helical computerized tomography for the evaluation of patients with acute flank pain. J Urol.

[12] Smith RC, Rosenfield AT, Choe KA, Essenmacher KR, Verga M, Glickman MG (1995). Acute flank pain: comparison of non-contrast-enhanced CT and intravenous urography. Radiology.

[13] Katz DS, Lane MJ, Sommer FG (1996). Unenhanced helical CT of ureteral stones: incidence of associated urinary tract findings. AJR Am J Roentgenol.

[14] Katz DS, Scheer M, Lumerman JH, Mellinger BC, Stillman CA, Lane MJ (2000). Alternative or additional diagnoses on unenhanced helical computed tomography for suspected renal colic: experience with 1000 consecutive examinations. Urology.

[15] Mindelzun RE, Jeffrey RB (1999). The acute abdomen: current CT imaging techniques. Semin Ultrasound CT MR.

[16] Kellow ZS, MacInnes M, Kurzencwyg D, Rawal S, Jaffer R, Kovacina B (2008). The role of abdominal radiography in the evaluation of the nontrauma emergency patient. Radiology.

[17] MacKersie AB, Lane MJ, Gerhardt RT, Claypool HA, Keenan S, Katz DS (2005). Nontraumatic acute abdominal pain: unenhanced helical CT compared with three-view acute abdominal series. Radiology.

[18] Baker SR (1997). Unenhanced helical CT versus plain abdominal radiography: a dissenting opinion. Radiology.

[19] Eisenberg RL (2008). The role of abdominal radiography in the evaluation of the nontrauma emergency patient: new thoughts on an old problem. Radiology.

[20] Denton ER, Mackenzie A, Greenwell T, Popert R, Rankin SC (1999). Unenhanced helical CT for renal colic–-is the radiation dose justifiable?. Clin Radiol.

[21] Kalra MK, Prasad S, Saini S, Blake MA, Varghese J, Halpern EF (2002). Clinical comparison of standard-dose and 50% reduced-dose abdominal CT: effect on image quality. AJR Am J Roentgenol.

[22] Hamm M, Knopfle E, Wartenberg S, Wawroschek F, Weckermann D, Harzmann R (2002). Low dose unenhanced helical computerized tomography for the evaluation of acute flank pain. J Urol.

[23] Kluner C, Hein PA, Gralla O, Hein E, Hamm B, Romano V (2006). Does ultra-low-dose CT with a radiation dose equivalent to that of KUB suffice to detect renal and ureteral calculi?. J Comput Assist Tomogr.

[24] Poletti PA, Platon A, Rutschmann OT, Verdun FR, Schmidlin FR, Iselin CE (2006). Abdominal plain film in patients admitted with clinical suspicion of renal colic: should it be replaced by low-dose computed tomography?. Urology.

[25] Preston DL, Shimizu Y, Pierce DA, Suyama A, Mabuchi K (2003). Studies of mortality of atomic bomb survivors. Report 13: Solid cancer and noncancer disease mortality: 1950–1997. Radiat Res.

[26] Brenner D, Elliston C, Hall E, Berdon W (2001). Estimated risks of radiation-induced fatal cancer from pediatric CT. AJR Am J Roentgenol.

